# Fellowship of the Royal College of General Practitioners by Assessment

**Published:** 1990-12

**Authors:** Denis Pereira Gray

**Affiliations:** General Practitioner, Exeter Director, Postgraduate Medical School, University of Exeter


### Fellowship of The Royal College of General Practitioners by Assessment

**Denis Pereira Gray, OBE, MA, FRCGP**
General Practitioner, Exeter
Director, Postgraduate Medical School, University of Exeter

In all the medical Royal Colleges Fellowship is a higher grade than membership of the College, but in the great majority of Royal Colleges Fellowship is awarded by election to a minority of the members who have been judged by the Council of the College, or by a relevant sub-committee, to have achieved professional distinction. This is the arrangement in the Royal Colleges of Physicians, General Practitioners, Pathologists, Psychiatrists and the newer Colleges of Ophthalmology and Anaesthetics.

The surgeons have always been somewhat different because the FRCS was established on the basis of an examination in the nineteenth century and membership of the Royal College of Surgeons of England has formed part of the conjoint examination for over 100 years. The surgeons have therefore always been committed to ensuring Fellowship on the basis of an examination and have therefore had a different system from the other Colleges throughout this century. There are however now suggestions that they may change their system so that they move more towards the arrangement that has evolved in all the other Royal Colleges.

In 1989 the Royal College of General Practitioners introduced an entirely new route to its Fellowship which was in addition to the previously established route. The new route was based on the principle that the purpose of the College is to raise standards of care for patients and that therefore entry to the highest grade of membership of the College should be seen to be related to good quality of care for patients. After a gestation period of over ten years, in which the ideas were discussed and considered, a steadily increasing drive towards Fellowship by assessment occurred in the last three years, largely as a result of major initiatives by doctors from the South West of England.

Working in parallel, central committees of the College, especially the Fellowship by Assessment Working Group within the Education Division, were strongly stimulated by the two faculties of the College in the South Western Region, the Tamar and Severn Faculties, both of which made major contributions. Tamar, led by its Officers, Geoffrey Smerdon, then Provost, Robert Sibbald, then Chairman, and Richard Parrott, Honorary Secretary, worked, through a Fellowship Nominations Committee, which met regularly and did an enormous amount of work in devising objective criteria on which to base a practice visit.

The principal contribution from the Northern end of the region, in the Severn Faculty, was through Richard Baker, who chaired the RCGP's Fellowship by Assessment Working Group for one of the three years concerned and greatly strengthened the documentation by referencing the criteria both to the scientific literature and to the formally adopted policies of the College. Meanwhile the whole process was given strong support from the Chairman of Council of the College, who for the first time came from the South West Region.

Fellowship by assessment represents a radical break from the previous arrangements in British medicine. First it is open to every member of the College of any age of 5 years' continuous standing, so it is a big step towards democratising the College and making Fellowship fair, open and widely available. Secondly, the standards concerned are agreed nationally by representatives from all parts of the United Kingdom and represent a consensus on what is "the highest possible standards of general medical practice", which is the aim of the College expressed in its Royal Charter.

This is a dynamic system whereby the criteria are reviewed every year so that they can be adjusted in the light of experience and new findings from research.

The actual process heavily devolves responsibility to the faculties of the College. In the South West, Severn covers the counties Gloucester, Avon and Somerset, and Tamar covers Devon and Cornwall. With the encouragement and advice of a colleague within the faculty, the applicants submit detailed evidence showing how they can meet the criteria. They are then visited in their own practices by a team of three peers, all of whom are general practitioners in active practice; one of these is nominated as the leader from the headquarters of the College, and the other two are local practitioners. The final process involves approval by the College's Fellowship Committee, followed by approval by the Council of the College, and finally comes election at an Annual General Meeting.

Visits take about half a day and the College is prepared to reimburse the assessors their travelling and locum costs. This makes the process quite expensive and the cost to applicants is £400.

Fellowship by this route can be seen as an aiming point. It sets a direction for new and old members of the College so that they can work towards it over several years.

It is fitting that having been so involved in the establishment of such a system the South West Region should be well represented among the first doctors to achieve this new award. The first from the West was Dr Tony Lewis of West Cornwall, who has subsequently moved to another practice in Exmouth and is now a Lecturer in General Practice at the Department there, and the most recent success has been Dr Ian Kerr, who works in a big group practice in Tiverton. Thus of the six first general practitioners to achieve this standard, the West Country has two. The main impact of the arrangement is not, however, likely to be on the small number of doctors who are presently passing it but on the much wider number of members of the College who are now working towards it in their practices, often planning to do it over many years because the standards are so demanding.

In several parts of the South Western Region general practitioners are meeting in groups stimulated by the faculties of the College and there are currently groups meeting in Cornwall (Robert Grundy, St Colomb) and Devon (Nicholas Bradley, Exeter), and groups are also being planned in Severn and Plymouth. This development does make Fellowship by assessment a truly educational process and it is leading to much discussion about criteria, the reasons for them, and the best ways of achieving them in practice.

Fellowship by assessment can be seen as a radical move in developing standards within a profession and an important step towards ensuring professional independence and self government. The history of this system, the present criteria, and the reasons for them in relation to College policy have all recently been published by the College as Occasional Paper 50, *Fellowship by Assessment*. This document is not restricted to College members and is generally available, price £7.50, from RCGP Enterprises Ltd, 14 Princes Gate, Hyde Park, London SW7 1PU.

